# Application of Computational Biology and Artificial Intelligence Technologies in Cancer Precision Drug Discovery

**DOI:** 10.1155/2019/8427042

**Published:** 2019-11-11

**Authors:** Nagasundaram Nagarajan, Edward K. Y. Yapp, Nguyen Quoc Khanh Le, Balu Kamaraj, Abeer Mohammed Al-Subaie, Hui-Yuan Yeh

**Affiliations:** ^1^School of Humanities, Nanyang Technological University, 14 Nanyang Dr, Singapore 637332; ^2^Singapore Institute of Manufacturing Technology, 2 Fusionopolis Way, Singapore 138634; ^3^Department of Neuroscience Technology, College of Applied Medical Sciences, Imam Abdulrahman Bin Faisal University, Jubail 35816, Saudi Arabia; ^4^Department of Clinical Laboratory Sciences, College of Applied Medical Sciences, Imam Abdulrahman Bin Faisal University, Dammam, Saudi Arabia

## Abstract

Artificial intelligence (AI) proves to have enormous potential in many areas of healthcare including research and chemical discoveries. Using large amounts of aggregated data, the AI can discover and learn further transforming these data into “usable” knowledge. Being well aware of this, the world's leading pharmaceutical companies have already begun to use artificial intelligence to improve their research regarding new drugs. The goal is to exploit modern computational biology and machine learning systems to predict the molecular behaviour and the likelihood of getting a useful drug, thus saving time and money on unnecessary tests. Clinical studies, electronic medical records, high-resolution medical images, and genomic profiles can be used as resources to aid drug development. Pharmaceutical and medical researchers have extensive data sets that can be analyzed by strong AI systems. This review focused on how computational biology and artificial intelligence technologies can be implemented by integrating the knowledge of cancer drugs, drug resistance, next-generation sequencing, genetic variants, and structural biology in the cancer precision drug discovery.

## 1. Introduction

Personalized or precision cancer therapy involves the identification of anticancer medicine for individual tumor molecular profiles, clinical features, and associated microenvironment of cancer patients [1, 2]. Precision medicine also aims to treat cancer more effectively with less adverse effects. According to a report by the International Agency for Research on Cancer (IARC), approximately 18.1 million of new registry on cancer cases and 9.6 million cancer-related deaths have been reported worldwide in 2018 [3]. Combined with classical cancer treatment methods, recent innovations in cancer treatment such as targeted chemotherapy, antiangiogenic agents, and immunotherapy were adapted by physicians on a case-to-case basis for better results [4]. In a number of instances, cancers such as hepatocellular carcinoma, malignant melanoma, and renal cancer often show intrinsic resistance to drugs without prior dosage of anticancer drugs [5]. In other cases, the initial response to the chemotherapy is remarkable. However, such a period is followed by a poor outcome, as cancer responds well to chemotherapy initially but later shows resistance due to development of resistance. Millions of cases regarding adverse drug resistance in cancer treatments are reported every year, which translates to a possibility of thousands of avoidable deaths. Such a dire situation thus calls for the designing of potential drugs. However, it is a time-consuming and complex process since each cancer patient responds differently to chemotherapy agent and its harmful effects are often unpredictable [6].

Ultimately, there is a crucial need to identify the primary mechanism with an ability to predict resistance to cancer therapies. The incorporation of tumor genetic profiling into clinical practice has improved the existing knowledge regarding the complex biology of tumor initiation and progression. Next-generation sequencing (NGS) is a platform commonly utilized by researchers to decode the genetic pattern of cancer patients, which allows for precision antitumor treatment based on their respective genomic profiles. It is clear that NGS plays a major role in treating diseases; however, it faces many technical challenges in its implementation. The highly accurate data obtained from NGS lead to the identification of a large set of genomic variations, in order to further identify the harmful variations of diseases. As such, specific modern computational algorithms are required to analyze and interpret the data. A number of computational tools have been developed to analyze the dataset that are integrated with genomic sequence and biochemical data on genetic polymorphism. Such tools will allow the prediction of functional consequences of deleterious polymorphism. Most of the tools were design followed by the combination of physicochemical properties of amino acids, protein structure information, and evolutionary sequence conservation analysis. Analyzing the functional consequence of genetic variation is not the limit; hence, directing such a analysis towards precision drug discovery and the structural attributes of drug interaction will bring about a new dimension in the cancer treatment. NGS technology usually produces huge set of data, and it is very difficult to analyze the data with the current existing tools. However, AI approaches have the capability to analyze NGS data in favor to identify suitable drug for individual patients.

Artificial intelligence (AI) proves to have an enormous potential in many areas of healthcare, including biomedical data analysis and drug discovery. The modern supercomputers and machine learning systems are able to explore the genetic data in order to identify the precision drugs. The key reason for applying AI in genetic data analysis is the completion of the human genome projects, which have reported huge amounts of genetic information. Over the last few years, the idea of using AI to accelerate precision drug identification to process and boost the success rates of pharmaceutical research programs has inspired a surge of activity in this area. Nowadays, biomedical studies can access extensive data sets due to the advancement of sequencing techniques and the accumulation of information on genetic variations. As such, there are currently greater prospects for precision medicine to come into the foreground of cancer treatment. As artificial intelligence makes use of the genetic profile for each patient, the right drug can be identified to cater to the patient's needs. Moreover, the artificial intelligence system is able to refine the key information in a short span of time. In this review, we aim to discuss about the integration of recent computational and biological techniques in order to develop a more effective cancer treatment. This will allow the fabrication of a precision drug identification platform through the application of artificial intelligence.

## 2. Literature Survey on Next-Generation Sequencing Technologies and Variant Calling Algorithms

In the early 1970s, a new technology was established to sequence the DNA molecule. However, its technical complexity, working cost, and limited availability of radioactive reagent made it difficult for the researchers to use this technology in the laboratory. Following this, the first-generation automated DNA sequence technology designed by Sanger and colleagues adopted a chain termination method [7]. Maiden et al. in 1990 used the DNA sequencing technology in the multilocus sequence-typing scheme for *Neisseria meningitidis* [8]. *Haemophilus influenzae* is the first environmental living microorganism that was sequenced in 1995 with the use of the Sanger sequencing methodology [9]. However, it is very expensive and time-consuming to sequence the whole human cell genome with this technology. In 1990, the human genome project was initiated with a goal to decode 3.2 billion base pairs of human genomes for biomedical research in disease diagnostic and treatment. Initially, the Sanger sequencing technology was used in this project worth 3.8 billion with international collaboration [10, 11]. Later in the early 2000s, another new technology emerged, namely, next generation sequencing (NGS) technology, which truly revolutionized the DNA sequencing process by reducing the time, cost, and labor. After 2010, genome sequencing was done on bacterial pathogens, which transfers the usage of technology from within the laboratory to public health practice. The sequencing technologies were used in several events of the critical infectious disease outbreak. Some examples include the cholera outbreak after a massive earthquake in Haiti during 2010 and the *E. coli* O104 : H4 disease outbreak, which was associated with consumption of fenugreek sprout in 2011 [12, 13]. In both cases, it was important to understand the virulent characteristic immediately, in order to reduce the progress of the disease, which will create massive morbidity and mortality. In these events, both academic and government research laboratories reacted quickly with NGS technology using crowd sourcing and open sharing of data. After these outbreaks, more public health laboratories have started to utilize NGS technology. Standardized NGS tests have been adopted in many countries' public laboratories for surveillance and in addition, NGS rated highly in specialized hospital laboratories [14, 15].

Between 1975 and 2005, the Sanger method was the predominant sequencing methodology. It has been considered as the gold standard for sequencing DNA that can produce 500–1000 bp long high-quality DNA reads. In 2005, 454 Life Science corporations introduced a revolutionized pyrosequencing technology referred to as “next generation sequencing (NGS) technology” [16]. This massive DNA sequencing technology is capable of reading and detecting thousand to millions of short DNA fragments in a single machine run without the need of cloning. Later versions of DNA sequencing technology were able to generate short reads (50–400 bp) and long reads (1–100 kb). The working mechanism and performance have been extensively discussed in many review articles [17, 18]. The MiSeq and MiniSeq technologies offer low to mid sample processing, moderate instrumentation cost and user-friendly working methods with automated and affordable cost per sample around $120 per 5 MB genome sequencing. Therefore, they have been the primary choice of technology for public health and disease diagnostic laboratories. The technologies HiSeq, NextSeq, and NovaSeq are considered as more suitable for core sequencing facility, irrespective of their high instrumentation cost since its cost per sample is low throughout the sequencing. However, they require automation for library preparation. By utilizing the full capacity of a sequencing machine, the cost can be effectively further reduced. In addition, the real-time testing is critical since the laboratory specific samples are sequenced in the laboratory-owned sequencing machines, which are highly tuned for the routine samples. For example, around 4000 isolates can be processed annually with a single MiSeq instrument and the use of v3 reagent, which would cover real-time testing in a laboratory. Amongst the NGS sequencing platforms, HiSeq as a product of Illumina generates the best quality of base call data. Ion Torrent, as a product of thermos fisheries, also performs sequencing by synthesis and its detection based on the hydrogen ions released during DNA polymerization that can be measured by the solid-state pH meter [19]. The PGM and S5 instruments are the IonTorrent equivalents for the Illumina MiniSeq and MiSeq; the ion proton is equivalent of Illumina NextSeq. The performance, the strength, and the weakness of prominent genomic sequencing platform have been compared and tabulated in Table 1.

Mutation/variation in the genetic code is considered as an important cause of cancer and thus it is the major focus in cancer research and treatment. The recent advancement in the sequencing technology can generate a huge set of data that can be explored by computational methods to identify the de novo mutation. Theoretically, all mutations including in the genomic region or variant allele frequency (VAF) can be identified with sufficient read depth. However, the noise in the files makes it difficult to identify them with confidence. A number of computational methods have been designed to identify the genetic variation or mutation from the complex DNA sequence reads (Table 2). The process involves a procedure with three features: read processing, mapping and alignment, and variant calling. As a first step, the read processing algorithms such as NGS QC Toolkit [20], Cutadapt [21], and FASTX Toolkit have been used to trim out the low quality and exogenous sequences such as sequencing adapter. During the library preparation of targeted sequencing, some of the protocol uses unique molecular identifiers (UMI) and PCR primers. In order to trim and remove the oligonucleotide, a customized read processing script must be developed. Second, the processed reads are mapped with the reference genome to identify the sequence, which is followed by base-by-base alignment. Most common applying, mapping, and alignment tools for DNA sequence include NovoAlign, BWA [22], and TMAP (for Ion Torrent reads) and as for RNA sequencing, splice-aware aligner tools such as STAR [23] and TopHat [24] are used. Genome Analysis Toolkits (GATKs) are the widely used tool for variant calling; following the procedures generally is important in this step such as PCR de-duplication, indel-realignment, and base quality recalibration [25, 26]. The final process is the variant calling, which is an important step for identifying correct variants/mutations from artifacts stemming from the prepared library, sequencing, mapping or alignment, and sample enrichment. A number of germ line and somatic variant calling tools have been developed which are freely available for analysis. The underlying knowledge is quite vary for somatic and germline variant calling tools. The rate of allele frequency in germline variants calling algorithms is expected to be 50 or 100%, and hence germline variant calling algorithms have accurately identified AA or AB or BB among these three genotypes, which fit the best [26–29]. Most artifacts occur in less frequency rate and are less likely to create a problem since in this case homozygous reference would be the most likely genotype. However, neglecting this type of artifact is not recommended in somatic variant calling because some original variants may also occur in very low frequencies in situations such as impure sample, rare tumor subclone, and in circulating DNA. Hence, the greatest challenge of the somatic variant calling algorithm is to accurately identify the low-frequency variants from artifacts, which can be done using advanced error correction technology and a more sensitive statistical model. Genetic variants can be classified into three major groups: insertion and deletion (indel), structural variant (such as duplication, translocation, copy number variation, etc.), and single nucleotide variant (SNV). Currently, only minimum number of variant caller algorithms is available to predict all these type variants, as they need specific trained algorithms. For single nucleotide variation and short indels (typically size ≤10 bp), the primary procedure is to check for nonreference nucleotide bases from the stack of sequence that cover each position. To evaluate the genotypic variants, mostly probabilistic modeling tools are used or to classify the artifact from the odds of variant. For structural variants and long indels, since the reads are too short to span over any variant, the focus is to identify the break points based on the patterns of misalignment with paired end reads or sudden change of read depth. Split reads assembly and de novo methods are frequently used for somatic variant analysis and long indel detection. GATK Unified Genotyper/Haplotype Caller, GAP, and MAQ are some of the tools used for germline variant calling [25, 26, 30, 31]. For somatic variant calling unified haplotype and genotype calling algorithms have been used, but the core algorithms are not formulated for this analysis following that it performs poorly for low-frequency somatic variants, and this information is highlighted in some independent studies as well as in the GATK documentation [32, 33]. Some other variant callers such as thunder and CRISP that are mainly used for pooled samples are also used for variant analysis [34].

## 3. Global Cancer Report

A reason for the majority of global deaths is the occurrence of noncommunicable diseases (NCDs) [35]. During the 21st century in almost every country of the world, cancer is the primary cause of deaths and this prevalent issue hinders the extension of life expectancy. In 2015, the World Health Organization (WHO) estimated that cancer is a dominant cause of mortality and morbidity before the age of 70 years in 91 of 172 countries, and in the rest of the 22 countries, it ranks as the third or fourth reason for death. Cancer morbidity and mortality are rapidly increasing worldwide. Ultimately, there are complex reasons such as the lack in the disease prevalence and distribution as well as an aging population. In addition, the population increase and its socioeconomic conditions serve as major causes of cancer death [36, 37]. Cancer incidence is mostly reported in developing countries, where the rising number of the disease is parallel by a modification in the genetic profile of common tumor genetic types. A serious observation made regarding the ongoing changes in the poverty-related and infection-related cancers is that they are increasingly common in some developed continents with the highest incomes, such as Oceania, Asia, North America, and Europe. The root cause of these cancers is often the modernized lifestyles [37–39]. However, the differing cancer tumor genetic profiles of various countries and even between specific ethnic zones signify that geographic variation still exists, with a persistence of local factors in populations at vastly different phases of economic and social transition. This is elucidated by the major differences in frequency of infection related to cancers, including stomach, liver, and cervix in the regions at opposite ends of the human development spectrum [38]. With regard to this information, a statistical analysis regarding the cancer burden worldwide in 2018 was made based on the GLOBOCAN 2018 observation of cancer morbidity and mortality analyzed by the International Agency for Research on Cancer (IARC) [40]. The same parameters as used in 2002 [41], 2008 [41], and 2012 [42] were taken into consideration to observe the cancer morbidity and mortality at the global level. As a result, an assessment has been made regarding the geographic differences observed across twenty predefined global regions. In the total number of cases, 11.6% lung cancer has been observed and as for the total number of cancer-related deaths, 18.4% were cause of lung cancer. For females, breast cancer is the next most common cancer at 11.6% followed by colorectal cancer at 10.2% and prostate cancer at 7.1% for incidence. As for mortality, the prominent causes are colorectal cancer at 9.2% followed by both liver and stomach cancer at 8.2%. In males, lung cancer is the most commonly occurring cancer and the primary reason for cancer mortality. In addition, prostate and colorectal cancers are the leading causes for incidence of cancer and liver and stomach cancer for cancer-related deaths. In the female population, breast cancer is the most commonly occurring cancer and the primary reason for cancer death followed by colorectal and lung cancer for incidence. Next to these former reasons, cervical cancer ranks fourth for both morbidity and mortality. Over 65% of newly identified cancer morbidity and mortality is caused by top ten cancer types worldwide observed.

## 4. Complication in Cancer Drug Discovery

From the beginning of human civilization, there has been a long history of drug discovery and development. The discovery and development of drugs is still a time-consuming process, whereby around 10–15 years needed to bring a single effective drug from the laboratory to market. Moreover, it requires huge investments, averaging from US$500 million to $2 billion [43, 44]. The high cost of drug development will probably affect the ability of patients with financial limitations to acquire the treatment. The expenditure to treat cancer in the USA will expect to rise from $124.57 billion in 2010 to $157.77 billion by 2020 [45]. In addition to discovery and development, drug production needs to fulfill satisfactory levels of toxicity, efficacy, and pharmacodynamics and pharmacokinetic profiles of the potential drugs candidate in in vitro and in vivo studies. In addition, preclinical studies were conducted to examine the efficacy and safety of the drug in humans in four different phases. Basically, drug development is hindered by a high rate of failure regarding their toxicity and efficacy profiles. According to the recent reports, even though new drug candidates exhibit high safety profile in Phase I trials, most of the drugs results fail due to poor efficacy in Phase II clinical trials [46]. Compared with other processes of drug discovery, oncology-related therapeutic discovery has the highest failure rate in clinical trials. Recent development in cancer treatment allows for the discovery of target specific drugs. However, only 1 of every 50K to 100K target specific anti-cancer drugs is approved by the US FDA. Furthermore, only 5% of anticancer drugs getting into Phase I clinical trials are often approved [47]. The target-specific anticancer drugs approach failed and it is still being investigated by oncologists to understand the underlying molecular mechanism. From the investigation reports, it is understood that in the development of cancer, more than 500 signaling molecules have been contributed [48]. However, the target-based drug discovery mostly focuses on inhibiting the identified signaling molecules. An investigation has to be made further in examining the drug-gable targets other than the reputed signaling molecules. Most of the drug targets are classified based on the preclinical studies; however, most prefindings are not exactly replicable in the clinical treatment. The number of potential drugs such as olaparib and iniparib showed promising results in preclinical stages. However, these preclinical in vitro and in vivo studies do not exactly consider the human cancer microenvironment [49–51]. In addition, the lack of quality in the pharmacodynamics and pharmacokinetics examination of drugs results in failure. Further poor testing strategies also majorly impact the drug's potential to translate from the preclinical findings to the medical treatment [52].

## 5. Cancer Drug Resistance

Drug resistance can be attributed to the decrease in the drug potency and efficacy to produce its desired effects. It stands as a big obstruction to treatment of the disease and affects the overall survival of the patient. Notably, local or locoregional, as well as distant tumor metastases leading in the paradox of therapy-induced metastasis (TIM), can result in resistance to anticancer treatments [5, 53, 54]. In a number of cases, tumors such as hepatocellular carcinoma, malignant melanoma, and renal cancer frequently show intrinsic resistance to anticancer drugs even without prior exposure to chemotherapy, resulting in a poor response during the initial stages of the treatment [5]. In some other cases, a chemotherapy agent may initially show its desired outcome. However, it is often followed by a poor response with harmful side effects due to the emergence of acquired drug resistance. So far, radiotherapy and surgery are the possible treatment methods for the removal of cancer cells. More systemic treatments are required to treat metastatic tumors or hematologic malignancies. Current forms of implementing systemic treatment are target-specific chemotherapy, immunotherapy, and antiangiogenic agents [53]. In most cases, drug resistance develops due to acquired and/or intrinsic genetic modulations. Intrinsic resistance may be induced by (a) modification of function and/or expression of the drug target, (b) drug breakdown, (c) changes in the drug carrying mechanism between the cellular membrane, (d) changes in the drug binding efficiency/efficacy with its binding target [54, 55]. Nuclear receptors and ATP-dependent membrane transporters are the primary factors that mediate the intrinsic cellular resistance [56]. Furthermore, cellular metabolic pathway systems, such as ceramide glycosylation, decrease the efficacy of anticancer drugs [57]. In addition, improved DNA damage repair mechanism increases drug resistance by reducing influx, increasing efflux, inhibiting drug accumulation through cell membrane transporters, and inactivating drugs [58, 59]. In reports of recent studies, the primary anticancer drugs had started to show signs of resistance against the known targets such as *TP53* [60]. Moreover, acquired drug resistance induced by environmental and genetic factors that enhance the development of drug resistant tumor cell or induce mutations of genes involved in relevant metabolic pathways [61, 62].

## 6. Computational Methods for Variant Classification

In recent days, the genetic mechanism behind human disease can be understood by next-generation sequencing technology approaches such as whole exome sequencing (WES) [63, 64]. Through WES sequencing technology, the genetic variants in the human genome can be detected. So far, several reports have documented that missense variants are the major cause of genetic diseases [65, 66]. However, not all the missense variants are involved in human genetic diseases as only deleterious variants are associated with Mendelian diseases, cancers, and undiagnosed diseases [67]. Identifying all deleterious variants through experimental validation is quite complicated work since it would require large amounts of labor and resources. Hence, computational methods have been developed to address this problem effectively by adopting different approaches like sequence evolutionary, sequence homology, and protein structural similarity [68–87]. Commonly there are three methods of prediction: (i) Sequence conservation methods, which generally note the degree of nucleotide base conservation at a particular position in comparison with the multiple sequence alignments information. (ii) Protein function-prediction methods that calculate the chance of a missense variant creating structural modification that affect protein function. (iii) Ensemble methods that integrate both sequence and structural information to calculate the effect of deleterious variants. In most cases for the missense variant identification tool development, all these methods have been adopted [88–90] and those tools are utilized in our studies [91–94]. VarCards is a database developed with the information on classified human genetic variants [95, 96]. It has integrated the functional consequences of allele frequencies, different computational methods, and other clinical and genetic information associated with all possible coding variants [97]. However, it is still difficult to understand the variance in performance of the computational methods, which differ under different conditions. Different studies have compared the performance of the missense variant prediction computational methods; however, they have not made use of the experimentally evaluated and considered benchmark datasets [98–103]. Particularly, these studies focus on assessing the receiver operating characteristic (ROC) curves. However, other parameters such as accuracy, specificity, sensitivity, and area under the curve (AUC) were not completely evaluated. There might be cases whereby geneticists and clinicians use computational tools to predict the harmful variants among the missense variants during the genetic counseling for known disease causing genes [104]. Hence, it is expected that these tools have to distinguish the pathogenic variants with a high-sensitivity rate [87]. In addition, VEST3 [78], REVEL [85], and M-CAP [87] are some recently developed algorithms that were not completely assessed in the previous studies. However, a recent study compared 23 computational pathogenicity prediction tools such as (i) ten function-prediction methods: fitCons [81], FATHMM [88], LRT [70], Mutation Taster [75], Mutation Assessor [76], PolyPhen2-HVAR [73], PolyPhen2-HDIV [73], SIFT [72], PROVEAN [77], and VEST3 [78]; (ii) four conservation methods: PhastCons [68], phyloP [69], GERP++ [74], and SiPhy [71]; and (iii) nine ensemble methods: DANN [83], CADD [79], Eigen [86], GenoCanyon [82], FATHMMMKL [84], MetaLR [80], M-CAP [87], REVEL [85], and MetaSVM [80]. The pathogenicity prediction scores of the 23 methods can be downloaded from the dbNSFP database v3.3 [105]. These predicted scores have been commonly used in medical genetics to identify the deleterious variant from the benign. Furthermore, prediction scores and other clinical information and genetic information were used alongside the VarCards [97] database. The cutoff values used to identify the deleterious missense variants were observed from ANNOVAR [106], dbNSFP database [105], and the original studies.

## 7. Artificial Intelligence in Precision Drug Discovery

The National Institute of Health (NIH) highlighted that precision medicine is an emerging strategy for disease prevention and treatment, which considers the individual variation in the gene, lifestyle, and environment [107]. This strategy helps researchers and doctors to prevent and treat the disease more accurately based on the genetic profile of the individuals. To make the strategy more comprehensive, it requires powerful supercomputer facilities and creative algorithms that can independently learn in an unprecedented way from the trained set of data. Artificial intelligence uses the cognitive ability of physicians and biomedical data for further learning to produce results. Artificial intelligence is broadly classified into three categories: artificial general intelligence, artificial narrow intelligence (ANI) and artificial super intelligence [108]. ANI is still in a stage of development and is expected to hit the market in by the next decade. ANI also has the caliber to deeply analyze the data set, find new correlation, draw conclusion, and support physicians. Well-established pharmaceutical companies have started to use the deep learning, super computers, and ANI in precision drug discovery process. Physicians may use the deep learning algorithms in many areas of disease diagnosis and treatment like oncology [109], dermatology [110], cardiology [111], and even in neurodegenerative disorders. However, developing such algorithms is crucial and critical in terms of exploring the knowledge of a physician in synchronizing with the algorithm development. Deep learning aims to identify unique genetic patterns in large genomic data sets and medical records and consequently identify genetic variations/mutations and their association with various diseases. A system of biological approach combined with artificial intelligence can form new algorithms that are able to monitor the changes inside the cell upon genetic modulation in the DNA [112]. Drug development is a highly complicated process that requires a huge amount of time and finances. However, in clinical trials, most of the drugs are rejected due to toxicity and lack of efficacy. Making the process faster and more cost-effective will have a tremendous impact on modern-day health care and how innovations made in drug discovery. Atomwise is the biopharma that uses an artificial intelligence-integrated supercomputing facility to analyze the database's information on small molecular structures. With the AI facility, Atomwise has launched a program to identify medicine to treat the Ebola virus. Through the AI technology, the company has found two better drugs, which are more promising in killing Ebola virus. Without such AI technology, such a drug discovery would take several years, however, with the AI system will doing it in less than one day [113]. Although the use of AI might seem promising in the discovery of drugs, these pharmaceutical companies will need to prove the safety and potential of their method with peer-reviewed research. In continuation of this short summary, the role of artificial intelligence methodologies in genetic variant/mutation identification from genetic data, virtual screening of small molecules, and molecular dynamics simulation programs has been elaborated under the appropriate subheading.

### 7.1. Artificial Intelligence Methods Applied to Identify Variants/Mutations from Genetic Data

The aim of predictive models built based on machine learning approaches to draw conclusions from a sample of past observations and to transfer these conclusions to the entire population. Predicted patterns can be in different formats, such as nonlinear, linear, graph, cluster, and tree functions [114–116]. The machine-learning working mechanism is generally classified under four steps: filtering, data preprocessing, feature extraction, and model fitting and model evaluation. Supervised or unsupervised learning approaches are the two methods used in machine learning models. In supervised method to train the model, a known set of genetic information is required (for example, the start and end of the gene, promotors, enhancers, active sites, functional regions, splicing sites, and regulatory regions) in order to set the predictive models. This model is then used to find new genes that are similar to the genes of the training dataset. Supervised methods can only be used if a known training dataset of genetic codes available. Unsupervised methods are used if we are interested in finding the best set of unlabelled sequences that explain the data [117]. Machine learning methodologies have a wide range of application areas, and one of the most important applications is the identification of genetic variants and mutations [114, 118]. The machine learning approach called convolutional neural networks (CNNs) applied to the identification of genetic variants and mutations. The recently developed software's Torracina and Campagne analyzed genomic data to identify genetic variants/mutations and indel's using CNN method. Compared to previous methods [119], CNNs can substantially improve the performance in variant identifications [120]. Recurring variants in the genome content can be efficiently identified by means of this method [120, 121]. In the CNN method, the genetic sequence is analyzed as a 1D window using four channels (A,C,G,T) [122]. Genomic data used in machine learning models are classified under three categories 60% as training data, 30% as model testing data, and 10% as model validation data. Deep Variant is the recent method developed by Popolin et al. [123] for SNPs and indel detection with prediction precision >99% (at 90% recall). Deep sequence is the software used to identify the mutations [124], which also uses latent variables (a model using a decoder and an encoder network to predict the input sequence).

### 7.2. Applications of Artificial Intelligence in the Identification of Drugs

The virtual screening pipeline has been developed to reduce the cost of high throughput screening and further to increase efficiency and predictability in optimizing the potential small molecule [125, 126]. The strong generalization and learning process and machine-learning methods implementing aspects of AI models have been successfully implemented in different stages of the virtual screening pipeline. Virtual screening can be classified into two types: ligand- and structure-based virtual screening and with the former corresponding to situations wherein structural information from ligand-receptor binding is utilized and the latter to situations with its absence. Knowing the depth of the application of AI methods in virtual screening, we discussed the new findings in structure-based virtual screening driven by such approaches.

Advanced structure-based virtual screening methods have been developed with the help of potential AI algorithms based on nonparametric scoring functions. The correlation between the contributions to protein-ligand binding free energy and the feature vectors is implicitly observed through a data-driven manner from existing experimental data, which should enable the extraction of meaningful nonlinear relationships to obtain generalizing scoring functions [127–129]. The RF-based RF-score [128], SVM-based ID-score [130], and ANN-based NNScore are the AI-based non-predetermined scoring functions that have been developed to identify potential ligands with high accuracy rate. The recent advanced AI-based non-predetermined scoring methods outperform well in comparison with classical approaches in binding affinity predictions that have been discussed in several reviews [131–133].

In order to improve the scoring function performance, most of the AI techniques adopted the five major algorithms, namely, SVM, Bayesian, RF, deep neural network, and feed-forward ANN approaches. Ballester et al. reviewed the importance of machine learning regression algorithms in the enhancement of AI-based non-predetermined scoring functions to provide better binding affinity prediction between protein-ligand complexes. Based on the study, Ballester et al. developed a RF-based software to predict the protein-ligand docking score [134, 135]. Some other RF-based scoring functions such as B2B score [136], SFC score RF [137], and RF-IChem [138] have been developed to calculate the docking scores. On comparing with the above-listed tools, RF-score predictions are outstanding and thus it has been included with the istar platform, which involved large-scale protein-ligand docking [139]. SVM-based automated pipeline has been developed, capitalizing on the known weakness and strength of both ligand- and structure-based virtual screening.

For instance, from a pool of 18 million compounds to predict the novel c-Met tyrosine kinase inhibitors, Xie et al. [140] designed and used combined docking and SVM-based method. PROFILER is the automated workflow designed by Meslamani et al. [141] to identify the perfect targets having the highest probability of binding with bioactive compounds. PROFILER integrates with two structure-based approaches (protein-ligand-based pharmacophore searching and docking) and four ligand-based approaches (support vector regression affinity prediction, SVM binary classification, three-dimensional similarity search, and nearest neighbor affinity interpolation). In structure-based virtual screening, RF-score have been applied and performed well in identifying the targets. RF-Score-VS is the enhanced (DUD-E) scoring function that was trained on the full directory of useful decoy data sets (a set of 102 targets was docked with 15,426 active and 893,897 inactive ligands) [142].

The integration of AI techniques with structure-based virtual screening methods is the promising idea in the prediction of likely potential ligands. The AI technology has been adopted to improve the postprocessing process after the structure-based virtual screening process by reconsidering the scoring process calculated with docking algorithms using machine-learning models, with or without a consensus scoring. For example, AutoDock Vina can be incorporated with RF-Score-VS-enhanced method to get better performance in the virtual screening. The integration of advanced machine learning algorithms and automated ligand screening can help bring down the number of false positive and false negative predictions. Future work in this area is expected to consider physicochemical properties and structural information of the target protein.

### 7.3. Enhanced Molecular Dynamics Simulations with Artificial Intelligence

Computational chemistry is a potential technology to explore biochemical and structural behaviours of interest in a wide range of environments. Molecular dynamics simulations combined with multiscale molecular or quantum mechanics methods to measure the atomic level movement of a biomolecular system have been predominantly used to understand the behavior of molecules in recent studies [143–145]. However, it is too difficult to analyse the movement of large groups of atom in a stretch, and it requires powerful computational facilities. Integration of AI technology and computational chemistry can complete the high volume of simulation in an efficient way [146–148]. An established example is the construction of neural network potentials for high-dimensional systems with the Behler–Parinello symmetry function to asses thousands of atoms [149–151]. Many scientifically intensified problems have been explored recently such as solvation for Schrodinger equation [152], machine-learned density functional development [153–156], classification of chemical trajectory data, predictions of the molecular properties prediction of the excited state electrons [157, 158], many-body expansions [159], classification of chemical trajectory data [160], high-throughput virtual screening to identify novel materials [161–166], heterogeneous catalysts [167], and band gap prediction [168, 169].

Many advancements have been made in this field, such as introduction of reweighting correction to calculate the output at an estimated level of theory with high precision (for example: quantum chemistry methods) based on the output predicted at an inexpensive baseline theory level (for example: semiempirical quantum chemistry), which has been examined for the estimation of thermochemical properties of active molecules [170] and more recently in the calculation of free energy changes during chemical reactions [171]. Even though it is a challenging task to combine AI algorithms and computational chemistry to explore the chemical datasets in order to identify the potential drug candidates in high magnitude of time, the molecular mechanics/quantum mechanics inspired artificial intelligence developers will likely be widely used to speed up the process while keeping quantum mechanical precision. This technical combination truly supporting AI approaches become a live technique in drug discovery.

## 8. Summary and Outlook

**Figure 1:**
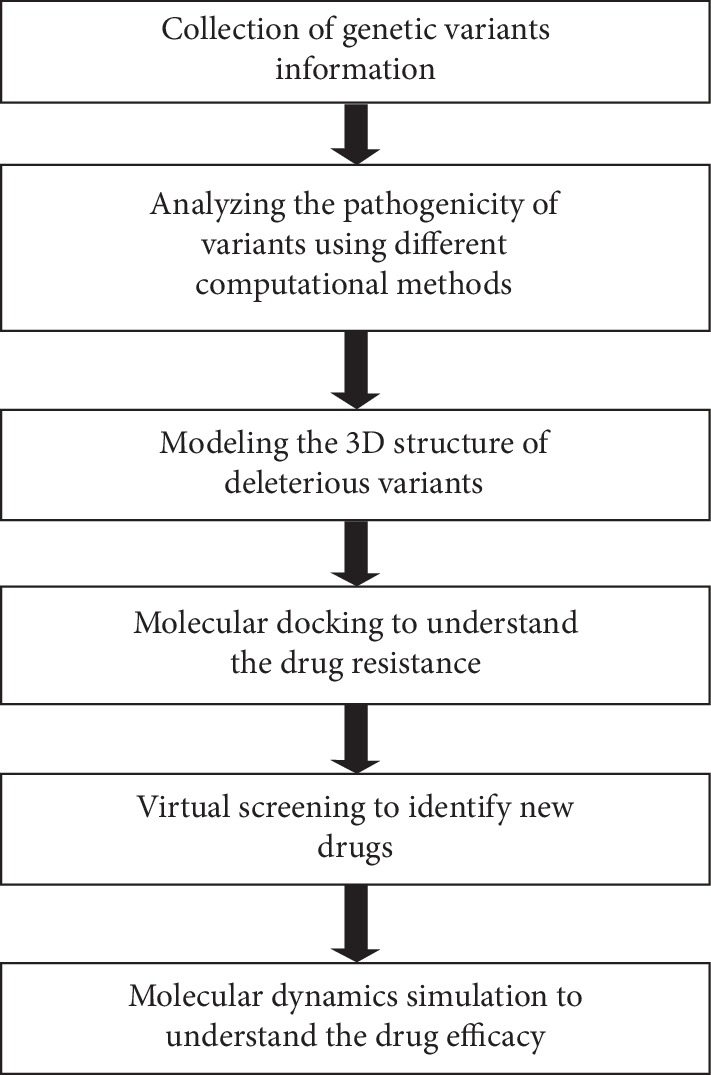
Computational pipeline to analyze the variants and to identify the precision drugs.

New targeted drugs for cancer treatment have to be developed to overcome cellular chemotherapy resistance and in addition must have the potential to inhibit “hub” genes. The primary role of those identified drugs is to achieve the highest therapeutic effect by eliminating tumor cells, with less adverse effects. Understanding the underlying mechanisms of the patient's responses to cancer drugs and the unravelling of their genetic code would lead to the identification of new precision therapies that may improve the patient's overall health and quality of life. Classical methods employed in the discovery of drugs are time- and cost-consuming. In response, computational biology has the efficiency to identify the precision drugs quickly. Current computational tools and software have an impact on the different phases of the drug discovery process. A number of studies have been performed by utilizing different computational approaches to identify the precision drugs that are suitable to particular genetic variant/s [91–94]. The methodology combined with the collection of genetic variants, prediction of pathogenicity using various computational tools, modeling the protein three-dimensional structure with particular variant/s, molecular docking of standard drug with variant/mutant structures, virtual screening to identify the specific drug, and performing molecular dynamics simulation allow for a better understanding of the efficacy of the drug (Figure 1). However, one limitation of the adopted methodology was that all the steps have been performed manually. It is necessary to bring radical change in the current computational methodology in order to identify precision drugs. We have shown in this review how artificial intelligence and computational biology approaches can be integrated to identify and discover cancer precision medicines.

**Figure 2:**
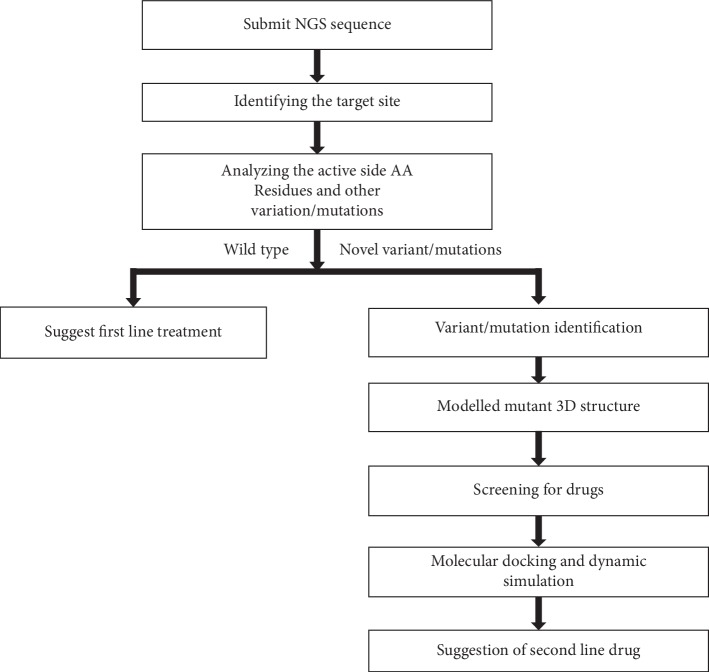
Suggested pipeline for cancer precision drug discovery.

Artificial intelligence integrated with computational biology has the potential to change the way drugs are designed and discovered. This approach was initially implemented at the Chapel Hill Eshelman School of Pharmacy at the University of North Carolina. The system is known as Reinforcement Learning for Structural Evolution, and it is well known by its acronym ReLeaSE. It is the computer software involving a set of algorithms incorporated with two neural networks programs, which can be considered to fulfill both roles of a student and a teacher. The teacher knows the linguistic rules and the syntax, which underlies the vocabulary of about 1.7 million known biologically active small molecules. Having been trained by the teacher, the student will understand the process over time and eventually become adept at finding the potential molecules that could be considered for developing new drugs. AI also positively influences precision medicine. The traditional drug discovery process of analyzing small data sets focused on a particular disease is offset by AI technology, which can rationally discover and optimize effective combinations of chemotherapies based on big datasets. The AI systems are built based on the experimental results and does not involve mechanistic hypotheses or any predictive models. Further, artificial intelligence technology can be applied in various ways such as to identify biomarkers, develop better diagnoses, and identify novel drugs. However, one important application of artificial intelligence lies in finding target-based precision drugs. As we can see, artificial intelligence has acquired a key role in shaping the future of the health sector. An automated integrated system, involving the analysis of genetic variants by deep/machine learning methods, molecular modeling, high throughput structure-based virtual screening, molecular docking, and molecular dynamics simulation methods, will enable rapid and accurate identification of precision drugs (Figure 2). Developing an AI-based system will indeed be beneficial in the drug discovery process and in the discovery of cancer precision medicine.

## Figures and Tables

**Table 1:** Comparison of performance, strengths and weaknesses of promising sequencing platforms.

Platform\instrument	Throughput range (Gb)	Read length (bp)	Strength	Weakness
Sanger sequencing				
ABI 3500/3730	0.0003	Up to 1 kb	Read accuracy and length	Cost and throughput

Illumina				
MiniSeq	1.7–7.5	1 × 75 to 2 × 150	Low initial investment	Run and read length
MiSeq	0.3–15	1 × 36 to 2 × 300	Read length, scalability	Run length
NextSeq	10–120	1 × 75 to 2 × 150	Throughput	Run and read length
HiSeq (2500)	10–1000	1 × 50 to 2 × 250	Read accuracy, throughput, low per sample cost	High initial investment, run length
HiSeq 3000/HiSeq 4000	105–1500	2 × 50 to 2 × 150	Read accuracy, throughput, low per sample cost	High initial investment, run and read length
NovaSeq 5000/6000	2000–6000	2 × 50 to 2 × 150	Read accuracy, throughput, low per sample cost	High initial investment, run and read length

IonTorrent				
PGM	0.08–2	Up to 400	Read length, speed	Throughput, homopolymers
S5	0.6–15	Up to 400	Read length, speed, scalability	Homopolymers
Proton	10–15	Up to 200	Speed, throughput	Homopolymers
Ion GeneStudio S5 prime System (ion 550″ chip)	10–50	Up to 200 (2 runs in one day)	Read length, speed, scalability	Homopolymers

Oxford nanopore				
MInION	0.1–1	Up to 100 kb	Read length, portability	High error rate, run length, low throughput
GridION X5	50–100	Up to 1000 kb	The GridION X5 offers real time, long-read, high-fidelity DNA and RNA sequencing.	High error rate
Pacific BioSciences				
PacBio RSII	0.5–1	Up to 60 kb(Average 10 kb, N50 20 kb)	Read length, speed	High error rate and initial investment, low throughput
Sequel	5–10	Up to 60 kb(Average 10 kb, N50 20 kb)	Read length, speed	High error rate
Sequel II	9–13	Up to 160 Gb	Read length, speed	Initial investment

**Table 2:** List of tumor-normal somatic SNV callers and single-sample somatic and germline SNV callers sorted in alphabetical order.

Variant caller	Type of core algorithm	Type of variant	Type of variant caller
BAYSIC	Machine learning (ensemble caller)	SNV	Tumor-normal somatic SNV callers
CaVEMan	Joint genotype analysis	SNV	Tumor-normal somatic SNV callers
deepSNV	Allele frequency analysis	SNV	Tumor-normal somatic SNV callers
EBCall	Allele frequency analysis	SNV, indel	Tumor-normal somatic SNV callers
FaSD-somatic	Joint genotype analysis	SNV	Tumor-normal somatic SNV callers
FreeBayes	Haplotype analysis	SNV, indel	Tumor-normal somatic SNV callers
HapMuC	Haplotype analysis	SNV, indel	Tumor-normal somatic SNV callers
ISOWN	Supervised learning	SNV	Single-sample somatic and germline SNV caller
JointSNVMix2	Joint genotype analysis	SNV	Tumor-normal somatic SNV callers
LocHap	Haplotype analysis	SNV, indel	Tumor-normal somatic SNV callers
LoFreq	Allele frequency analysis	SNV, indel	Tumor-normal somatic SNV callers
LoLoPicker	Allele frequency analysis	SNV	Tumor-normal somatic SNV callers
MutationSeq	Machine learning	SNV	Tumor-normal somatic SNV callers
MuSE	Markov chain model	SNV	Tumor-normal somatic SNV callers
MuTect	Allele frequency analysis	SNV	Tumor-normal somatic SNV callers
OutLyzer	Noise level estimation	SNV	Single-sample somatic and germline SNV caller
Platypus	Haplotype analysis	SNV, indel, sv	Tumor-normal somatic SNV callers
Pisces	Poisson model on read count	SNV, indel	Single-sample somatic and germline SNV caller
PoreSeq	Nanopore specific	SNV, indel	Single-sample somatic and germline SNV caller
qSNP	Heuristic threshold	SNV	Tumor-normal somatic SNV callers
RADIA	Heuristic threshold	SNV	Tumor-normal somatic SNV callers
Seurat	Joint genotype analysis	SNV, indel,sv	Tumor-normal somatic SNV callers
SAMtools	Joint genotype analysis	SNV, indel	Tumor-normal somatic SNV callers
Shimmer	Heuristic threshold	SNV, indel	Tumor-normal somatic SNV callers
SNooPer	Machine learning	SNV, indel	Tumor-normal somatic SNV callers
SNVSniffer	Joint genotype analysis	SNV, indel	Tumor-normal somatic SNV callers
SOAPsnv	Heuristic threshold	SNV	Tumor-normal somatic SNV callers
SomaticSeq	Machine learning (ensemble caller)	SNV	Tumor-normal somatic SNV callers
SomaticSniper	Joint genotype analysis	SNV	Tumor-normal somatic SNV callers
Strelka	Allele frequency analysis	SNV, indel	Tumor-normal somatic SNV callers
Shearwater	Noise level estimation	SNV	Single-sample somatic and germline SNV caller
SiNVICT	Poisson model on read count	SNV, indel	Single-sample somatic and germline SNV caller
SNVer	Allele frequency analysis	SNV, indel	Single-sample somatic and germline SNV caller
SNVMix2	Genotype analysis	SNV	Single-sample somatic and germline SNV caller
SomVarIUS	Noise level estimation	SNV, indel	Single-sample somatic and germline SNV caller
SPLINTER	Noise level estimation	SNV, indel	Single-sample somatic and germline SNV caller
TVC	Ion Torrent specific	SNV, indel, SV	Tumor-normal somatic SNV callers
VarDict	Heuristic threshold	SNV, indel, SV	Tumor-normal somatic SNV callers
VarScan2	Heuristic threshold	SNV, indel	Tumor-normal somatic SNV callers
Virmid	Joint genotype analysis	SNV	Tumor-normal somatic SNV callers
